# Plastic Pollution in the World's Oceans: More than 5 Trillion Plastic Pieces Weighing over 250,000 Tons Afloat at Sea

**DOI:** 10.1371/journal.pone.0111913

**Published:** 2014-12-10

**Authors:** Marcus Eriksen, Laurent C. M. Lebreton, Henry S. Carson, Martin Thiel, Charles J. Moore, Jose C. Borerro, Francois Galgani, Peter G. Ryan, Julia Reisser

**Affiliations:** 1 Five Gyres Institute, Los Angeles, California, United States of America; 2 Dumpark Data Science, Wellington, New Zealand; 3 Marine Science Department, University of Hawaii at Hilo, Hilo, Hawaii, United States of America; 4 Washington Department of Fish and Wildlife, Olympia, Washington, United States of America; 5 Facultad Ciencias del Mar, Universidad Católica del Norte, Coquimbo, Chile; 6 Millennium Nucleus Ecology and Sustainable Management of Oceanic Island (ESMOI), Coquimbo, Chile; 7 Centro de Estudios Avanzados en Zonas Áridas (CEAZA), Coquimbo, Chile; 8 Algalita Marine Research and Education, Long Beach, California, United States of America; 9 eCoast Limited, Raglan, New Zealand; 10 Departement Océanographie et Dynamique des Ecosystemes, Institut français de recherche pour l′exploitation de la mer (Ifremer), Bastia, Corsica, France; 11 Percy FitzPatrick Institute of African Ornithology, University of Cape Town, Rondebosch, South Africa; 12 School of Environmental Systems Engineering and Oceans Institute, University of Western Australia, Crawley, Perth, Australia; University of Connecticut, United States of America

## Abstract

Plastic pollution is ubiquitous throughout the marine environment, yet estimates of the global abundance and weight of floating plastics have lacked data, particularly from the Southern Hemisphere and remote regions. Here we report an estimate of the total number of plastic particles and their weight floating in the world's oceans from 24 expeditions (2007–2013) across all five sub-tropical gyres, costal Australia, Bay of Bengal and the Mediterranean Sea conducting surface net tows (N = 680) and visual survey transects of large plastic debris (N = 891). Using an oceanographic model of floating debris dispersal calibrated by our data, and correcting for wind-driven vertical mixing, we estimate a minimum of 5.25 trillion particles weighing 268,940 tons. When comparing between four size classes, two microplastic <4.75 mm and meso- and macroplastic >4.75 mm, a tremendous loss of microplastics is observed from the sea surface compared to expected rates of fragmentation, suggesting there are mechanisms at play that remove <4.75 mm plastic particles from the ocean surface.

## Introduction

Plastic pollution is globally distributed across all oceans due to its properties of buoyancy and durability, and the sorption of toxicants to plastic while traveling through the environment [1], [2], have led some researchers to claim that synthetic polymers in the ocean should be regarded as hazardous waste [3]. Through photodegradation and other weathering processes, plastics fragment and disperse in the ocean [4], [5], converging in the subtropical gyres [6]–[9]. Generation and accumulation of plastic pollution also occurs in closed bays, gulfs and seas surrounded by densely populated coastlines and watersheds [10]–[13].

The impact of plastic pollution through ingestion and entanglement of marine fauna, ranging from zooplankton to cetaceans, seabirds and marine reptiles, are well documented [14]. Adsorption of persistent organic pollutants onto plastic and their transfer into the tissues and organs through ingestion [15] is impacting marine megafauna [16] as well as lower trophic-level organisms [17], [18] and their predators [19], [20]. These impacts are further exacerbated by the persistence of floating plastics, ranging from resin pellets to large derelict nets, docks and boats that float across oceans and transport microbial communities [21], algae, invertebrates, and fish [22] to non-native regions [23], providing further rationale to monitor (and take steps to mitigate) the global distribution and abundance of plastic pollution.

**Figure 1:**
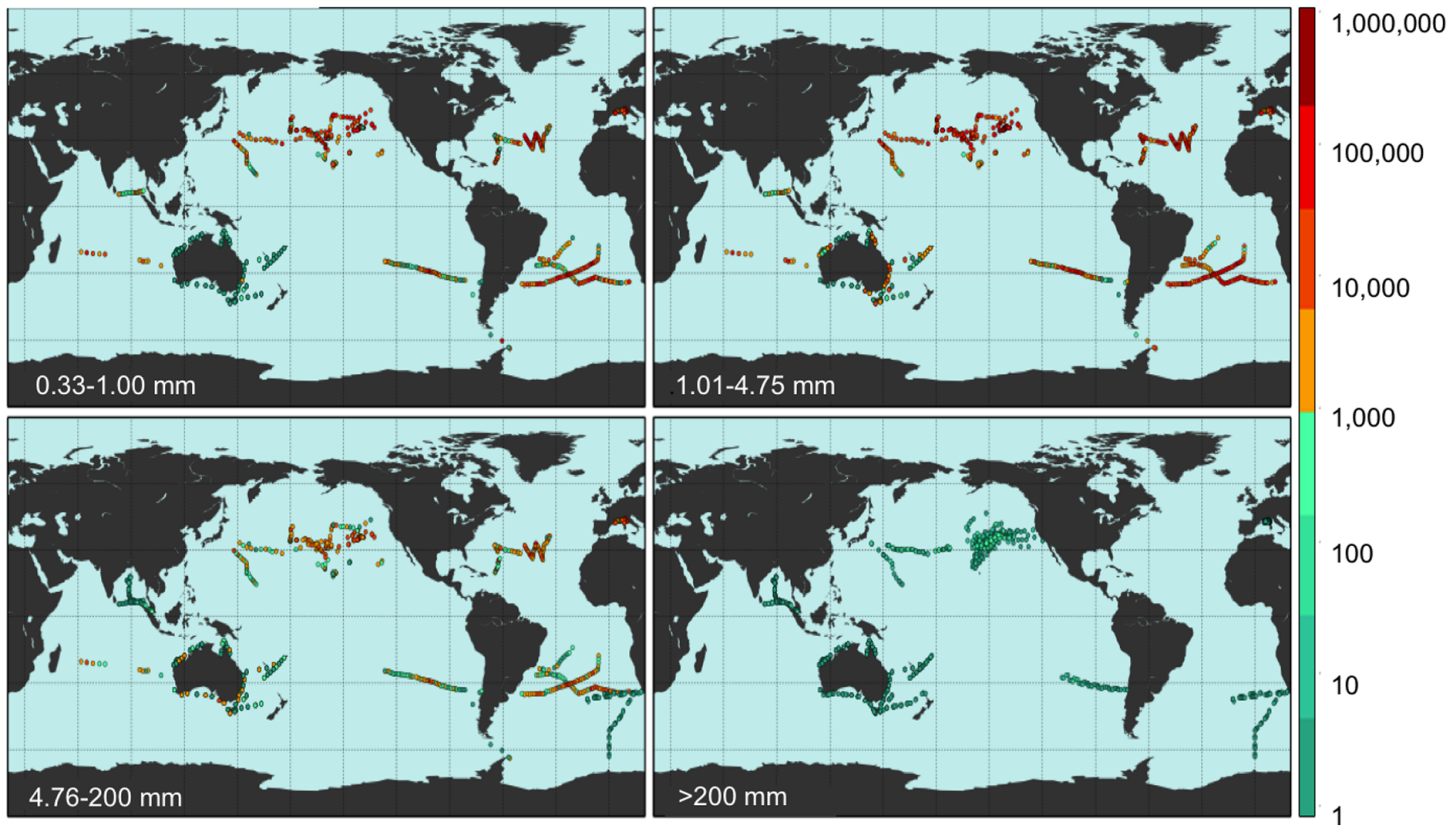
Field locations where count density was measured. Count density (pieces km^−2^; see colorbar) of marine plastic debris measured at 1571 stations from 680 net tows and 891 visual survey transects for each of four plastic size classes (0.33–1.00 mm, 1.01–4.75 mm, 4.76–200 mm, and >200 mm).

Despite oceanographic model predictions of where debris might converge [24] estimates of regional and global abundance and weight of floating plastics have been limited to microplastics <5 mm [19], [25]. Using extensive published and new data, particularly from the Southern Hemisphere subtropical gyres and marine areas adjacent to populated regions [7], [10], [13], [26], corrected for wind-driven vertical mixing [27], we populated an oceanographic model of debris distribution [28] to estimate global distribution and count and weight densities of plastic pollution in all sampled size classes. The oceanographic model assumes that amounts of plastic entering the ocean depend on three principal variables: watershed outfalls, population density and maritime activity. The dataset used in this model is based on expeditions from 2007–2013 (Table S1), surveying all five sub-tropical gyres (North Pacific, North Atlantic, South Pacific, South Atlantic, Indian Ocean) and extensive coastal regions and enclosed seas (Bay of Bengal, Australian coasts and the Mediterranean Sea), and include surface net tows (N = 680) and visual survey transects for large plastic debris (N = 891) totaling 1571 locations in all oceans (Fig 1). We also compared plastic pollution levels between oceans and across four size classes: 0.33–1.00 mm (small microplastics), 1.01–4.75 mm (large microplastics), 4.76–200 mm (mesoplastic), and >200 mm (macroplastic) (Fig. 1).

## Materials and Methods

### Net tow sample collection and analysis

Net tows were conducted using neuston nets with a standard mesh size of 0.33 mm towed between 0.5 and 2 m s^−1^ at the sea surface for 15–60 minutes outside of the vessel's wake to avoid downwelling of debris. Samples were preserved in 5% formalin. Using a dissecting microscope, microplastic was manually separated from natural debris, sorted through stacked Tyler sieves into three size classes [7], [10], [12], then counted individually and weighed together. During sample analysis the identity of smaller microplastics was confirmed with buoyancy and hardness tests. All items were counted and weighed to the nearest 0.01 mg. Using these data, trawl dimensions and distance traveled, count (pieces km^−2^) and weight (g km^−2^) densities were estimated. The slow tow speed and the washing of the net between the tows when needed provided sufficient confidence that any variation in sample collection efficiency due to the net size, difference in tow speed or tow time were negligible.

### Visual survey protocol

Visual survey transects of large plastic debris were carried out during expeditions to the South Pacific, North Pacific, South Atlantic, Indian Ocean, and waters around Australia, as well as part of the NOAA Trans-Pacific Marine Debris Survey in the North Pacific. Dedicated observers viewed the ocean surface on one side of the vessel out to 20 meters noting large debris items during timed observation periods [11], [13], [26], with start and stop positions used to calculate the area surveyed. Debris observations were broken into nine categories, four categories for fishing-related debris: buoy, line, net, and other fishing gear, and five categories for other plastics: bucket, bottle, foamed polystyrene, bag/film, or miscelaneous plastics (Table S2). Because observed debris cannot be collected and weighed, similar debris items in similar categories were collected from shorelines in northern-central Chile, South Africa, Atlantic coast of North America and the Hawaiian Archipelago to determine mean weights of items in the nine categories (Table S3). The two categories labeled ‘other fishing gear’ and ‘miscellaneous plastics’ were assigned a very conservative weight of 10 g per item. These mean weights were applied to visual survey transects to determine weight densities.

### Description of the model

Particle tracking is accomplished in two stages, first a hydrodynamic model describes oceanic circulation and second virtual particles are introduced into the flow field and allowed to move freely through hydrodynamic forcing. For this study, ocean surface currents are extracted from the oceanic circulation modeling system HYCOM/NCODA [29]. The HYCOM model is forced by the US Navy's Operational Global Atmospheric Prediction System (NOGAPS) and includes wind stress, wind speed, heat flux, and precipitation. The model provides systematic archiving of daily ocean circulation on a global scale with output data archived back to mid-2003. While the full HYCOM model contains 32 vertical layers, we only consider velocities in the surface layer as the principal driver of floating particles.

Velocity data extracted from HYCOM are then coupled to the Lagrangian particle-tracking model Pol3DD, which drives the dispersion of floating material. Pol3DD tracks and stores the origin, age, and trajectory information of individual particles [30]. Since wind driven currents are already expressed in the HYCOM hydrodynamic data, no additional wind stress terms were applied to the motion of particles. This model assumes that debris particles are mostly submerged in the water and extra forcing on potentially emerged parts of the debris is neglected.

### Model calibration using empirical data from 1571 locations

In this study we determined abundances and mass of microplastics starting at the lowest size of 0.33 mm, which is a commonly used lower limit for pelagic microplastics [31]. The prefixes micro, meso and macro in relation to plastic pollution are poorly defined. Generally accepted microplastic boundaries are based on typical neuston net mesh size (0.33 mm) and an upper boundary of approximately 5.0 mm [31]. We have used 4.75 mm as our upper boundary for microplastic because this is a size for standard sieves used for sample analysis in most of the expeditions contributing data to this manuscript. Mesoplastic has a lower limit of 4.75 mm, and no defined upper limit. In this current study we set the upper boundary of mesoplastic at 200 mm, which represents a typical plastic water bottle, chosen because of its ubiquity in the ocean. Macroplastic has no established lower boundary, though we set it at 200 mm, while the upper boundary is unlimited. There is a clear need for consistent measures in the field [31], and herein we followed a practical approach using commonly employed boundaries and logistic considerations (net and sieve sizes) in order to integrate an extensive dataset that covers the entire global ocean, including areas that have never been sampled before.

Of the 1571 field locations that contributed count data (Fig. 1), a total of 1333 stations also had weight data (Fig. S4). All these data were used to calibrate the numerical model prediction of plastic count and weight density [28]. For the comparison, we fit the model results to measured data by a linear system of equations of the form:
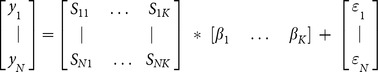


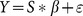
where y_i_ is the logarithm of a measured value of plastic count density (pieces km^−2^) or weight density (g km^−2^) for each of the N number of samples. K is the number of model output cases with s_ij_ a dimensionless model solution at the location of sample y_i_. β_k_ and ε_N_ are the computed weighting coefficients and the error terms for a particular dimensionless model solution s_ij_. This method can be used to fit an arbitrary number of model output cases to any number of measured data points producing a weighting coefficient and error term for each case.

In the model we used a set of three model results (K = 3), corresponding to different input scenarios [28]: urban development within watersheds, coastal population and shipping traffic. Values of β and ε are determined for both the concentration distribution (pieces km^−2^) and the weight distribution (g km^−2^) of each of the four size classes based on the linear system of equations. To compare the model results directly to the measured data, the weighting coefficient β_k_ computed above is used to scale the model output for each of the output scenarios.

### Adjusting estimated weight and count due to vertical distribution

Wind-driven mixing of the surface layer will drive particles downward, which causes underestimations of plastic in the ocean if relying on surface sampling only. We used a vertical distribution equation from Kukulka et al. [27], relating the ratio of the true number of particles/measured number of particles with the frictional velocity of water (u_*w_ = [*t/r_w_*]^1/2^, where *t* is the wind stress and *r_w_* is the density of water).

Our data from 680 net tows includes Beaufort Scale sea states, each with a wind speed range. Before using the vertical distribution equation, we transformed these data into wind stress values, by applying the Smith [32] coefficient for sea surface wind stress (N/m^2^) as a function of wind speed (m/s). These data were then used in the vertical distribution equation to adjust the total particle count of plastic for each station.

To estimate the increased mass due to vertical distribution, we attributed the same percentage increase in particle count to particle weight.

### Estimating expected particle counts based on fragmentation of large particles

We use conservative estimates of fragmentation rates to show that the model results of particle count in each size class differ substantially from our expected particle counts. To estimate fragmentation rates, we assumed that all particles, including the largest ones had a thickness of 0.2 mm. This assumption is conservative, because it is well known that many larger items have a wall thickness substantially larger than this. We assumed smaller particle sizes for the largest size classes, while for the smallest size class (0.33 mm–1.00 mm) we assumed a conservative particle diameter of 0.8 mm – this is substantially larger than most microplastics collected at the sea surface. Thus, our fragmentation estimates are highly conservative because for the macroplastics that generate plastic fragments we consider lower initial mass than commonly found at sea, while for the microplastics in our fragmentation exercise we consider larger particles than typically found at sea. Fragmentation of one macroplastic item (200 mm diameter) into typical mesoplastic fragments (50 mm diameter) would result in 16 particles, fragmentation of one 50 mm diameter mesoplastic item into typical large microplastics (2 mm diameter) results in 625 particles, and fragmentation of one large microplastic item (2 mm diameter) into small microplastics with a diameter of 0.8 mm results in 6.25 particles.

We then used these ratios in a stepwise approach to estimate particle counts in each size class based on the model results of particle count in the next-higher size category. For example, in the North Pacific the modeled data show 0.33×10^10^ particles in the macroplastic size class. Using our estimated fragmentation ratio of 1∶16 between macro and mesoplastic, we expect 5.33×10^10^ particles in the mesoplastic size class for the entire North Pacific. These fragmentation ratios between size categories are utilized to estimate the expected particle count for large and small microplastic particles. This stepwise approach is simplistic, because it assumes that the system is close to equilibrium. We recognize that rates of new plastic entering the ocean are unknown, as well as outputs of plastic due to beaching, sinking and mechanisms of degradation, and use these fragmentation estimates as first crude intent to reveal the dynamics of floating plastics in the oceans.

### Ethics Statement

During these sampling procedures, no permits were required as we only collected plankton samples, and those samples were collected in international waters.

## Results

Based on our model results, we estimate that at least 5.25 trillion plastic particles weighing 268,940 tons are currently floating at sea (Table 1). There was a good correspondence between the model prediction and measured data for particle count and weight (Figs. S1 and S2, Table S4). Our estimates suggest that the two Northern Hemisphere ocean regions contain 55.6% of particles and 56.8% of plastic mass compared to the Southern Hemisphere, with the North Pacific containing 37.9% and 35.8% by particle count and mass, respectively. In the Southern Hemisphere the Indian Ocean appears to have a greater particle count and weight than the South Atlantic and South Pacific oceans combined.

**Table 1:** Model results for the total particle count and weight of plastic floating in the world's oceans.

	Size class	NP	NA	SP	SA	IO	MED	Total
Count	0.33–1.00 mm	68.8	32.4	17.6	10.6	45.5	8.5	183.0
	1.01–4.75 mm	116.0	53.2	26.9	16.7	74.9	14.6	302.0
	4.76–200 mm	13.2	7.3	4.4	2.4	9.2	1.6	38.1
	>200 mm	0.3	0.2	0.1	0.05	0.2	0.04	0.9
	Total	199.0	93.0	49.1	29.7	130.0	24.7	525.0
Weight	0.33–1.00 mm	21.0	10.4	6.5	3.7	14.6	14.1	70.4
	1.01–4.75 mm	100.0	42.1	16.9	11.7	60.1	53.8	285.0
	4.76–200 mm	109.0	45.2	17.8	12.4	64.6	57.6	306.0
	>200 mm	734.0	467.0	169.0	100.0	452.0	106.0	2028.0
	Total	964.0	564.7	210.2	127.8	591.3	231.5	2689.4

Estimated total count (n×10^10^ pieces) and weight (g×10^8^ g; or g×10^2^ tons) of plastic in the North Pacific (NP), North Atlantic (NA), South Pacific (SP), South Atlantic (SA), Indian Ocean (IO), Mediterranean Sea (MED), and the global ocean (Total). Estimates were calculated after correcting for vertical distribution of microplastics [27].

Of the 680 net tows, 70% yielded density estimates of 1000–100,000 pieces km^−2^ and 16% resulted in even higher counts of up to 890,000 pieces km^−2^ found in the Mediterranean. The vast majority of these plastics were small fragments. Although net tow durations varied, the majority of all tows (92.3%) contained plastic, and those locations without plastic were outside the central areas of the subtropical gyres. This pattern is consistent with our model prediction that ocean margins are areas of plastic migration, while subtropical gyres are areas of accumulation. The 891 visual surveys revealed that foamed polystyrene items were the most frequently observed macroplastics (1116 out of 4291 items), while derelict fishing buoys accounted for most (58.3%) of the total macroplastic weight (Table S2). These observations are conservative, recognizing that items with marginal buoyancy, dark color and small size are more difficult to see, especially during challenging environmental conditions (depending on sea state, weather and sun angle).

**Figure 2:**
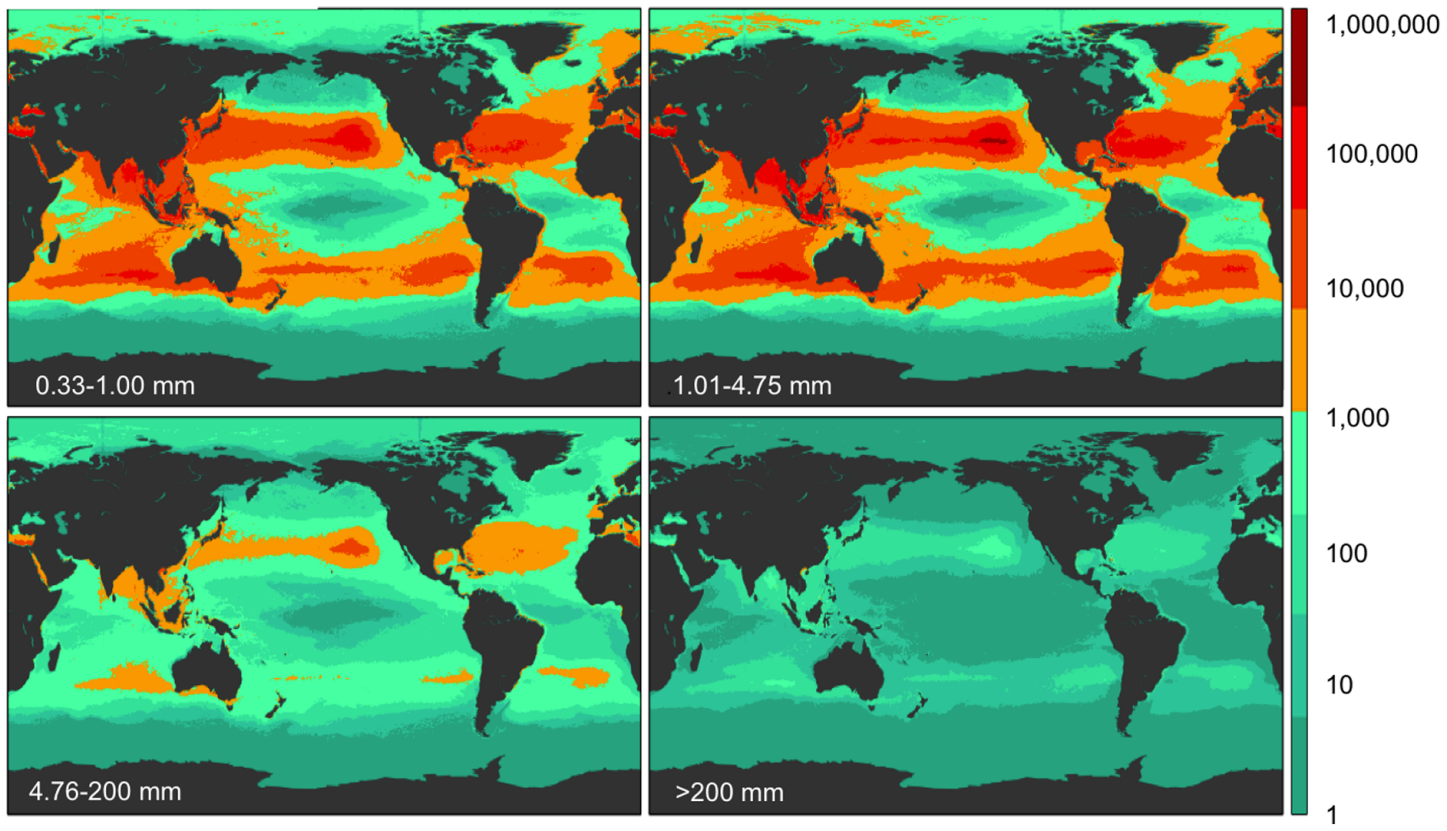
Model results for global count density in four size classes. Model prediction of global count density (pieces km^−2^; see colorbar) for each of four size classes (0.33–1.00 mm, 1.01–4.75 mm, 4.76–200 mm, and >200 mm).

**Figure 3:**
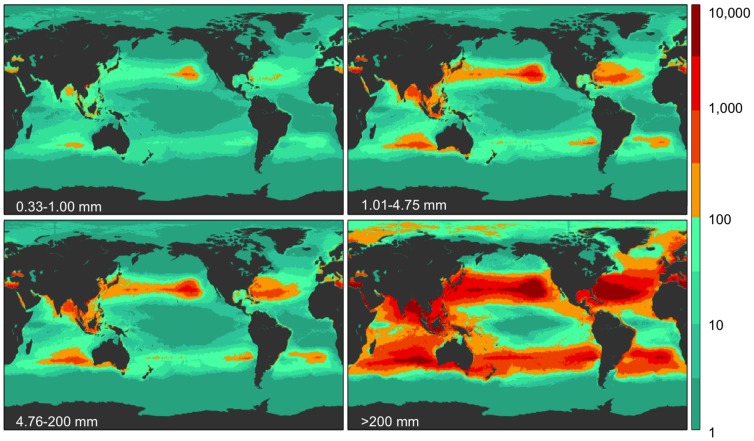
Model results for global weight density in four size classes. Model prediction of global weight density (g km^−2^; see colorbar) for each of four size classes (0.33–1.00 mm, 1.01–4.75 mm, 4.76–200 mm, and >200 mm). The majority of global weight is from the largest size class.

The data from the four size classes (small microplastics, large microplastics, meso- and macroplastics) were run separately through the model, producing four maps each for count and weight density (Figs. 2 and 3). The mean errors (ε) associated with these predictions can be seen in Table S5. Combining the two microplastic size classes, they account for 92.4% of the global particle count, and when compared to each other, the smallest microplastic category (0.33–1.00 mm) had roughly 40% fewer particles than larger microplastics (1.01–4.75 mm) (Table 1). Most small microplastics were fragments resulting from the breakdown of larger plastic items; therefore we expected the smallest microplastics to be more abundant than larger microplastics. We observed the opposite in all regions globally except in the S. Pacific where large and small microplastic counts were nearly equal.

The expected numbers of microplastics (large and small) were an order of magnitude larger than the data-calibrated model counts of microplastics in the world's oceans (Fig. S3). The expected numbers were derived from conservative estimates of fragmentation from macroplastic to smaller size classes. In contrast to the apparent dearth of microplastics mesoplastics were observed more frequently than expected by the fragmentation ration. For example, in the North Pacific the modeled data show 0.33×10^10^ particles in the macroplastic size class. Using our estimated fragmentation ratio of 1∶16 between macro and mesoplastic, we expect 5.33×10^10^ particles in the mesoplastic size class for the entire North Pacific. In this case our modeled data show 13×10^10^ mesoplastic particles, indicating our fragmentation rates underestimated the data-calibrated model results. This discrepancy could be due to lags in the fragmentation of buoyant mesoplastic and macroplastic, or because mesoplastic items, such as water bottles and single-use packaging, enter the ocean in disproportionate numbers when compared to macroplastic. However, the magnitude of the discrepancy between all size classes suggests that there is differential loss of small microplastics from surface waters.

We found a similar pattern of material loss from the sea surface when comparing the weight of the four size classes. The data showed the weight of plastic pollution globally was estimated to comprise 75.4% macroplastic, 11.4% mesoplastic, and 10.6% and 2.6% in the two microplastic size classes, respectively. Our data suggest that a minimum of 233,400 tons of larger plastic items are afloat in the world's oceans compared to 35,540 tons of microplastics.

## Discussion

This is the first study that compares all sizes of floating plastic in the world's oceans from the largest items to small microplastics. Plastics of all sizes were found in all ocean regions, converging in accumulation zones in the subtropical gyres, including southern hemisphere gyres where coastal population density is much lower than in the northern hemisphere. While this shows that plastic pollution has spread throughout all the world's oceans, the comparison of size classes and weight relationships suggests that during fragmentation plastics are lost from the sea surface. Simple comparisons across size classes allowed us to suggest possible pathways for oceanic plastics, and below we discuss these pathways and mechanisms involved.

Plastic pollution is moved throughout the world's oceans by the prevailing winds and surface currents. This had been shown for the northern hemisphere where long-term surface transport (years) leads to the accumulation of plastic litter in the center of the ocean basins [6], [7]. Our results confirm similar patterns for all southern hemisphere oceans. Surprisingly, the total amounts of plastics determined for the southern hemisphere oceans are within the same range as for the northern hemisphere oceans (Table 1), which is unexpected given that inputs are substantially higher in the northern than in the southern hemisphere [28]. This could mean that plastic pollution is moved more easily between oceanic gyres and between hemispheres than previously assumed [28], leading to redistribution of plastic items through transport via oceanic currents. Furthermore, there might also be important sources of plastic pollution in the southern hemisphere that had not been accounted for, such as currents from the Bay of Bengal that cross the equator south of Indonesia.

Alternatively, a large proportion of plastics might be lost from the sea surface, more so than considered by previous models, and these losses might be disproportionally higher in the northern hemisphere, leading to similar magnitudes in remaining plastic litter at the sea surface. Indeed, stranding of floating plastics on local seashores seems to be more important in the northern than in the southern hemisphere [28], [33]. Other losses (sinking, degradation) may also be responsible for the fact that northern hemisphere oceans contain relative plastic loads that are lower than expected based on global input scenarios. Herein we applied a correction for vertical distribution to all samples related to wind-driven turbulence [27]. Other hydrodynamic processes including downwelling at convergence zones may also influence the vertical distribution of slightly buoyant particles such as microplastics. We suggest that future sampling campaigns use the spatial distribution of sea surface features to better design their sampling efforts and come up with improved global plastic mass inventories.

Other estimates of global and regional weight of microplastic pollution are within the same order of magnitude as our estimates. A study using an 11-year data set in the North Pacific [9] estimates a weight of 21,290 metric tons of floating microplastic, and ours for the same region is 12,100 metric tons. A recent study on the global distribution of microplastic [25] suggests that the total floating microplastic load ranges between 7,000 and 35,000 metric tons, and ours is 35,500 metric tons. This study [25] also found a 100-fold discrepancy between expected microplastic weight and abundance and their observations, indicating a tremendous loss of microplastics. The similarities between our results and those of this study [25] gives us further confidence in our estimates and support our hypothesis that the ultimate fate of buoyant microplastics is not at the ocean surface.

The observations that there is much less microplastic at the sea surface than might be expected suggests that removal processes are at play. These include UV degradation, biodegradation, ingestion by organisms, decreased buoyancy due to fouling organisms, entrainment in settling detritus, and beaching [4]. Fragmentation rates of already brittle microplastics may be very high, rapidly breaking small microplastics further down into ever smaller particles, making them unavailable for our nets (0.33 mm mesh opening). Many recent studies also demonstrate that many more organisms ingest small plastic particles than previously thought, either directly or indirectly, i.e. via their prey organisms [34]–[36]. Numerous species ingest microplastics, and thereby make it available to higher-level predators or may otherwise contribute to the differential removal of small particles from the sea surface, e.g. by packaging microplastics into fecal pellets [37], thus enhancing sinking. Furthermore, there is increasing evidence that some microbes can biodegrade microplastic particles [38]–[40]. This process becomes more important as plastic particles become smaller since at decreasing particle size the surface area∶volume relationship is increased dramatically and oxidation levels are higher, enhancing their biodegradation potential. Thus, bacterial degradation and ingestion of smaller plastic particles by organisms may facilitate their export from the sea surface. In this manner, incorporation of smaller plastics into marine food chains could not only generate impacts on the health of the involved organisms [17]–[20], but also contribute to the removal of small microplastics from the sea surface [37].

Plastics Europe, a trade organization representing plastic producers and manufactures, reported that 288 million tons of plastic were produced worldwide in 2012 [41]. Our estimate of the global weight of plastic pollution on the sea surface, from all size classes combined, is only 0.1% of the world annual production.

However, we stress that our estimates are highly conservative, and may be considered minimum estimates. Our estimates of macroplastic are based on a limited inventory of ocean observations, and would be vastly improved with standardization of methods and more observations. They also do not account for the potentially massive amount of plastic present on shorelines, on the seabed, suspended in the water column, and within organisms. In fact, the larger weight of macroplastic relative to meso- and microplastic, and the global estimate of floating plastic weight relative to the weight of plastic produced annually, indicates that the sea surface is likely not the ultimate sink for plastic pollution. Though significant proportions of meso- and macroplastics may be stranding on coastlines (where some of it could be recovered), removal of microplastics, colonized by biota or mixed with organic debris, becomes economically and ecologically prohibitive, if not completely impractical to recover. This leaves sequestration in sediment the likely resting place for plastic pollution after a myriad of biological impacts along the way, thus reinforcing the need for pre-consumer and post-consumer waste stream solutions to reverse this growing environmental problem.

By generating extensive new data, especially from the Southern Hemisphere, and modeling the plastic load in the world's oceans in separate size classes, we show that there is tremendous loss of microplastics from the sea surface. The question “Where is all the Plastic?” [42] remains unanswered, highlighting the need to investigate the many processes that play a role in the dynamics of macro-, meso- and microplastics in the world's oceans.

## Supporting Information

Figure S1 **Comparison of mean and modeled densities.** Comparison of data and model predictions for count density (A - pieces km^−2^) and weight density (B - weight km^−2^) for four size classes from six ocean regions: North Pacific (NP), North Atlantic (NA), South Pacific (SP), South Atlantic (SA), Indian Ocean (IO), and Mediterranean Sea (MED).
(TIFF)

Figure S2 **Regression analysis of measured and modeled data.** Linear regression of modeled vs. measured values (with correction for vertical distribution) of plastic pollution in terms of count density (A - pieces km^−2^) and weight density (B - weight km^−2^) for each of the four size classes.
(TIFF)

Figure S3 **Comparison of modeled versus expected particle counts (n×10^10^ pieces) for the global oceans based on conservative fragmentation estimates.** The data-calibrated model results of particle count for the global oceans (see Table 1) in each size class differ substantially from conservative estimates of particle counts based on assumed fragmentation of the number if particles in the next-larger size category. We used simple estimates of particle sizes with 0.2 mm thickness and corresponding diameters, and fragmentation factors of 16 for breakdown of a 200 mm diameter particle into particles of 50 mm diameter, 625 for breakdown of a 50 mm diameter particle into particles of 2 mm diameter, and 6.25 for breakdown of a 2 mm particle into particles of 0.8 mm diameter.
(TIFF)

Figure S4 **Field locations where weight density was measured.** Weight density (g km^−2^) of marine plastic debris measured at 1333 stations from net tows and survey transects for each of the four size classes (0.33–1.00 mm, 1.01–4.75 mm, 4.76–200 mm, and >200 mm).
(TIFF)

Table S1 **Expeditions contributing field data.** 24 expeditions from 2007–13 contributed data collected at 1571 field locations, with count and weight data in four plastic size classes from six regions: North Pacific (NP), North Atlantic (NA), South Pacific (SP), South Atlantic (SA), Indian Ocean (IO), Mediterranean Sea (MED), and circumnaviating Australia (Au. Cirnav.). Locations marked with an asterisk indicate unpublished data and circles show the type of data collected at each expedition.
(TIFF)

Table S2 **Percent distribution of items from visual survey transects.** 4,291 macroplastic items (>200 mm) in nine categories were observed from all visual survey transects conducted in the North Pacific, South Pacific, South Atlantic, Indian Ocean, and Mediterranean Sea. Mean weights for macroplastic items (Extended Data Table 4) were used to determine percent weight distribution.
(TIFF)

Table S3 **Using beached macroplastic items to determine mean weight.** Mean weight of macroplastic items collected from coastal surveys in Chile (eastern S. Pacific), western South Africa (eastern S. Atlantic), east coast United States (western N. Atlantic), and the Hawaiian Islands, was applied to observed macroplastic items drifting in the ocean and then put through the model to calculate global weight densities. The two categories labeled ‘other fishing gear’ and ‘miscellaneous plastics’ were not calculated from weighing items, rather they were assigned a very conservative weight of 10 g.
(TIFF)

Table S4 **Comparison of measured to modeled means.** The measured means of regional count density (pieces km^−2^) and weight density (g km^−2^) of plastic in the North Pacific (NP), North Atlantic (NA), South Pacific (SP), South Atlantic (SA), Indian Ocean (IO), Mediterranean Sea (MED), are compared to modeled results. There is generally a good correspondence between the measured and modeled means for each region.
(TIFF)

Table S5 **Error margins from the linear regression.** Average error margin from the linear regression for the count density (pieces km^−2^) and weight density (g km^−2^) in the four size classes.
(TIFF)
