# Supplementary figures and images for: Plastic Pollution in the World's Oceans: More than 5 Trillion Plastic Pieces Weighing over 250,000 Tons Afloat at Sea
Source: PLoS One. 2014 Dec 10;9(12):e111913. doi: 10.1371/journal.pone.0111913 (PMC4262196; doi:10.1371/journal.pone.0111913)

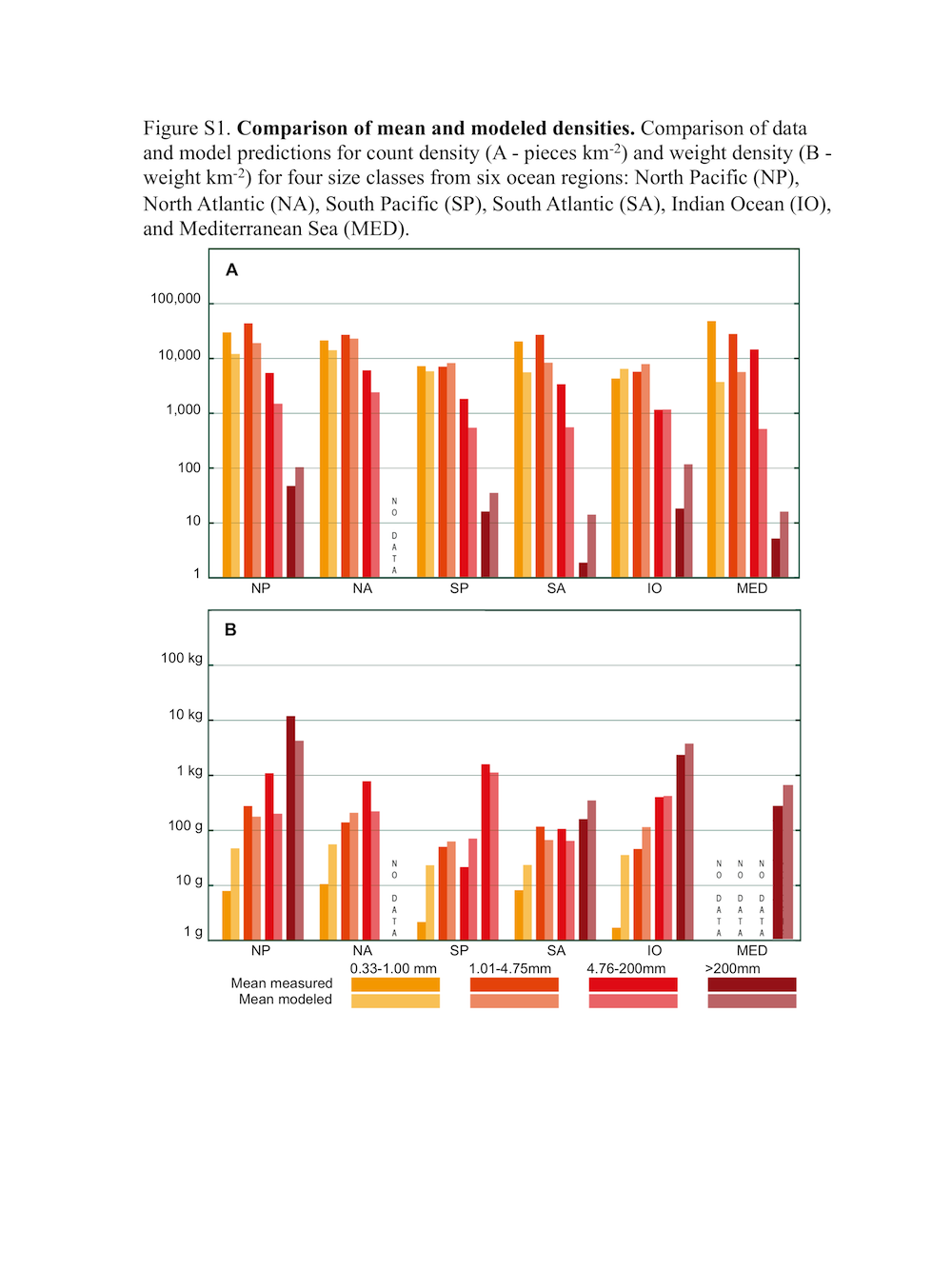

Supplement: Figure S1 — Comparison of mean and modeled densities. Comparison of data and model predictions for count density (A - pieces km−2) and weight density (B - weight km−2) for four size classes from six ocean regions: North Pacific (NP), North Atlantic (NA), South Pacific (SP), South Atlantic (SA), Indian Ocean (IO), and Mediterranean Sea (MED). (TIFF) [file pone.0111913.s001.tiff]

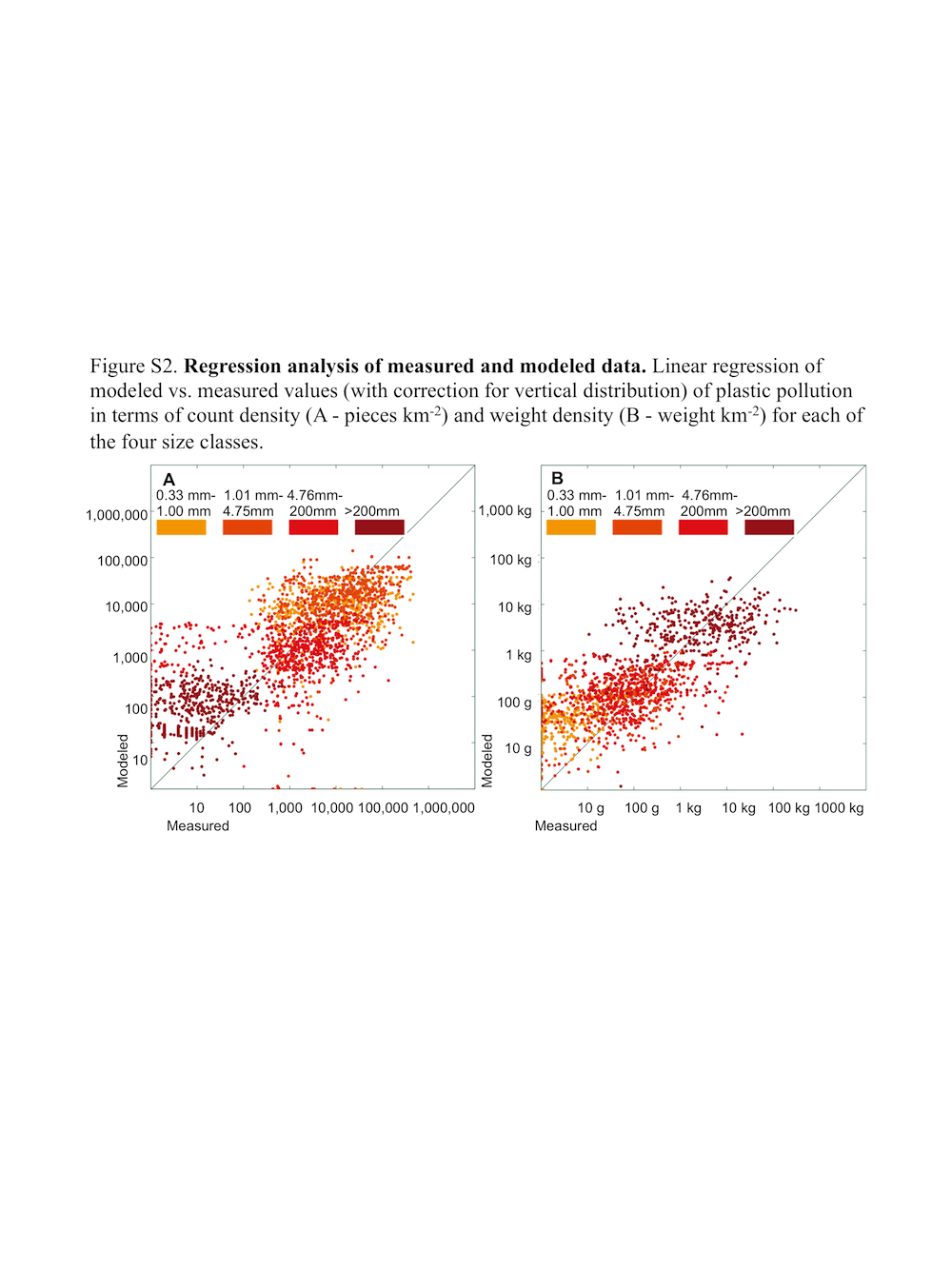

Supplement: Figure S2 — Regression analysis of measured and modeled data. Linear regression of modeled vs. measured values (with correction for vertical distribution) of plastic pollution in terms of count density (A - pieces km−2) and weight density (B - weight km−2) for each of the four size classes. (TIFF) [file pone.0111913.s002.tiff]

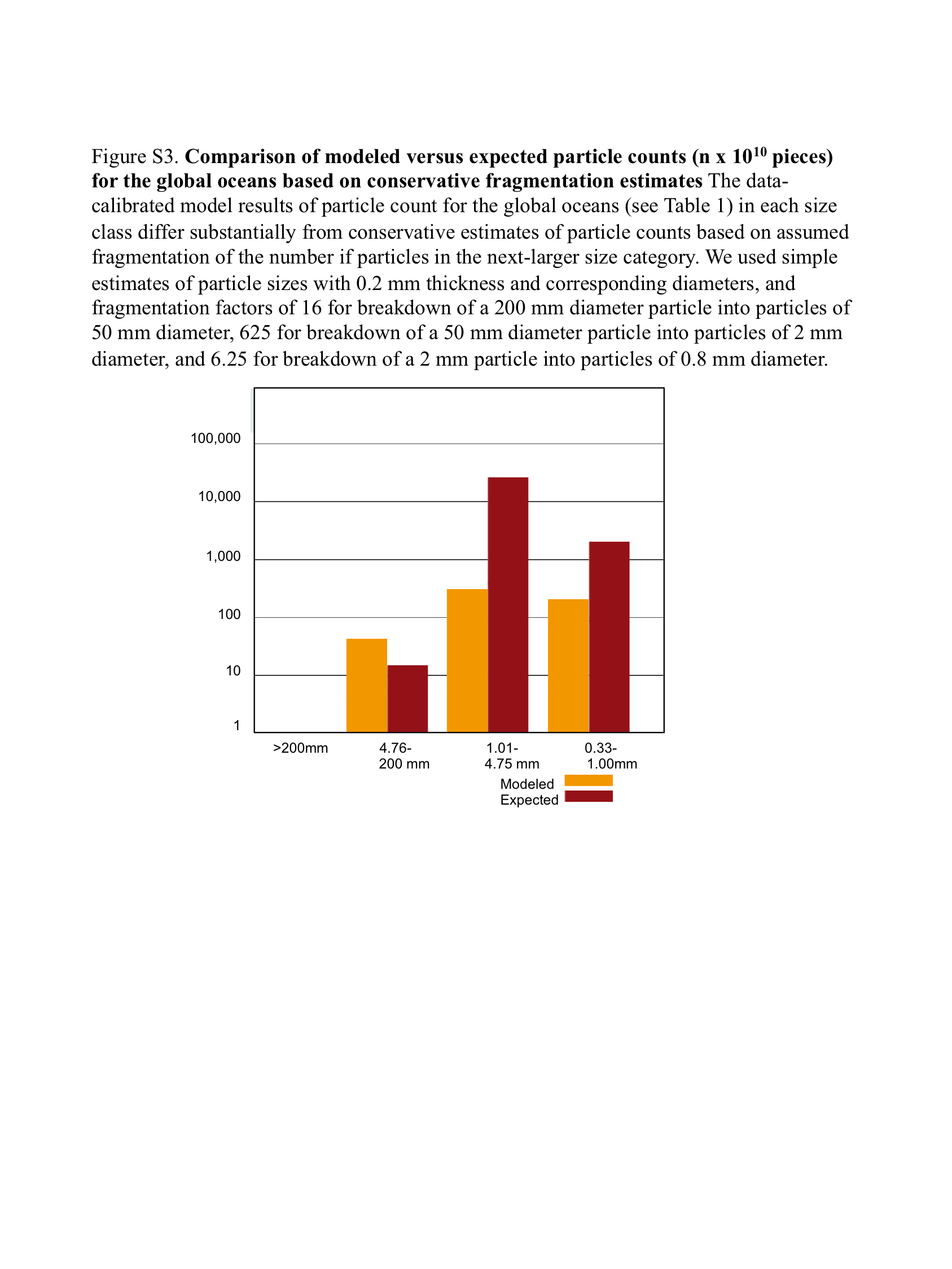

Supplement: Figure S3 — Comparison of modeled versus expected particle counts (n×1010 pieces) for the global oceans based on conservative fragmentation estimates. The data-calibrated model results of particle count for the global oceans (see Table 1) in each size class differ substantially from conservative estimates of particle counts based on assumed fragmentation of the number if particles in the next-larger size category. We used simple estimates of particle sizes with 0.2 mm thickness and corresponding diameters, and fragmentation factors of 16 for breakdown of a 200 mm diameter particle into particles of 50 mm diameter, 625 for breakdown of a 50 mm diameter particle into particles of 2 mm diameter, and 6.25 for breakdown of a 2 mm particle into particles of 0.8 mm diameter. (TIFF) [file pone.0111913.s003.tiff]

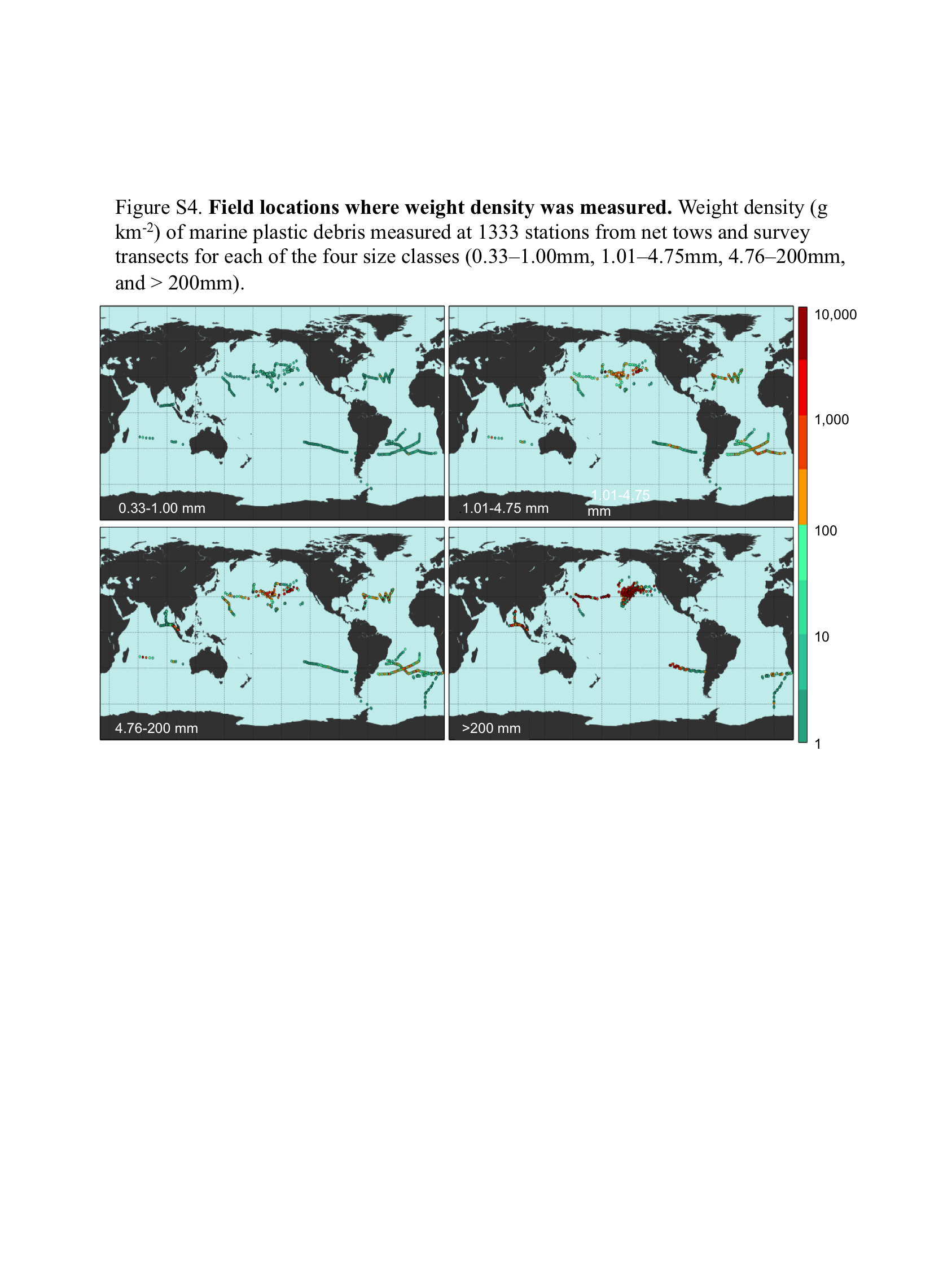

Supplement: Figure S4 — Field locations where weight density was measured. Weight density (g km−2) of marine plastic debris measured at 1333 stations from net tows and survey transects for each of the four size classes (0.33–1.00 mm, 1.01–4.75 mm, 4.76–200 mm, and >200 mm). (TIFF) [file pone.0111913.s004.tiff]
